# Superoxide dismutase and and its isoenzymes in children with juvenile idiopathic arthritis

**DOI:** 10.1186/1546-0096-12-S1-P158

**Published:** 2014-09-17

**Authors:** Henryka Mazur Zielinska, Michal Zielinski, Lukasz Pilarz, Dorota Karbowska, Ewa Birkner

**Affiliations:** 1 Department of Pediatrics, Zabrze, Medical University Of Silesia, Katowice, Poland; 2 Department of Biochemistry, Zabrze, Medical University Of Silesia, Katowice, Poland

## Introduction

Juvenile idiopathic arthritis (JIA) is not a rare disease in children. The etiology of the disease is unknown and various parameters are useful in establishing diagnosis. The research concerning reactive oxygen species in recent years shows that chronic oxidative stress may lead to chronic inflammation. Among the antioxidant systems present in cells superoxide dismutase is one of the preventive antioxidants i.e. enzymes reducing the number of generated free radicals.

## Objectives

The aim of the study was to examine the levels of superoxide dismutase and it’s isoenzymes in blood serum of JIA patients and healthy ones. Moreover levels’ changes in serum in children with inflammation of the joints depending on disease’s activity were investigated.

## Methods

The studied parameters were measured in blood serum of 59 patients with JIA, age from 2 to 18 years old, hospitalized in the Rheumatology Division of the Department of Pediatrics in Zabrze, in Silesian Medical University. The control group consisted of 25 healthy children.

## Results

The superoxide dismutase and it’s izoenzymes’ activity do not depend on JIA type.

Mn type superoxide dysmutase activity in acute phase in JIA patients is significantly higher in comparison to its activity in remission.

## Conclusion

Activity of superoxide dysmutase in blood serum in children with JIA depends on disease’s phase.

## Disclosure of interest

None declared.

